# Drug extravasation with Enfortumab vedotin

**DOI:** 10.1177/10781552231185505

**Published:** 2023-07-04

**Authors:** Christopher Ryan Grant, Dimitri de Kouchkovsky, Arash Rezazadeh Kalebasty, Nataliya Mar

**Affiliations:** 1 Department of Medicine, University of California Irvine Medical Center, Orange, CA, USA; 2 Department of Dermatology, University of California – San Diego, La Jolla, CA, USA; 3 Department of Hematology/Oncology, University of California Irvine Medical Center, Orange, CA, USA

**Keywords:** Drug extravasation, Enfortumab vedotin, antibody drug conjugate, irritant, vesicant, bladder cancer

## Abstract

**Introduction:**

Enfortumab vedotin is an antibody drug conjugate approved for management of pretreated locally advanced or metastatic urothelial carcinoma, which is associated with a rare risk of drug extravasation and soft tissue reactions.

**Case Report:**

We report two cases of EV extravasation with subsequent development of bullae and cellulitis.

**Management and Outcome:**

They were both treated for cellulitis and had conservative management without surgical intervention and were able to resume treatment with Enfortumab vedotin without subsequent adverse events.

**Discussion:**

We propose that EV acts as a vesicant upon extravasation, highlight measures to prevent extravasation events, and encourage appropriate measures when dealing such as attempt of aspiration, removal of catheter, application of compresses, and thorough documentation with photographic evidence.

## Introduction

Enfortumab vedotin (EV) is an antibody drug conjugate (ADC) approved in 2019 and used for management of pretreated locally advanced or metastatic urothelial carcinoma.^1^ The package insert for EV includes a rare risk of drug extravasation, which is defined as leakage of drug from a blood vessel into the surrounding tissue.^2^ Of 680 patients on clinical trials with EV, 0.3% developed extravasation reactions with secondary cellulitis, bullae or exfoliation, while an additional 1.6% experienced skin or soft tissue reactions.^1^ Antineoplastic agents can generally be classified as either neutrals, irritants, or vesicants, which influences management of subsequent extravasation complications. The mechanism of tissue damage caused by EV is poorly understood and guidance on management of drug extravasation and subsequent skin reactions is lacking. We report two cases of EV extravasation with subsequent development of skin and soft tissue sequela as well as discuss methods for prevention and management of this adverse event.

## Case # 1

A 59-year-old woman with history of recurrent non-muscle invasive urothelial bladder carcinoma refractory to Bacilli Calmette–Guerin (BCG) therapy was discovered to have multiple new infiltrative right renal masses and peritoneal lesions suspicious for metastasis on surveillance imaging. Biopsy of a renal mass showed atypical cells suspicious for urothelial carcinoma. The patient was deemed platinum-ineligible due to a glomerular filtration rate (GFR) of <30 mL/min and was initiated on pembrolizumab 200 mg every 21 days. Restaging CT imaging after two cycles of pembrolizumab demonstrated increased size of peritoneal lesions. Given the possibility of pseudoprogression, the patient was continued on pembrolizumab and EV 1.25 mg/kg on days 1, 8, 15 out of a 28-day cycle was added.

**Figure 1.**
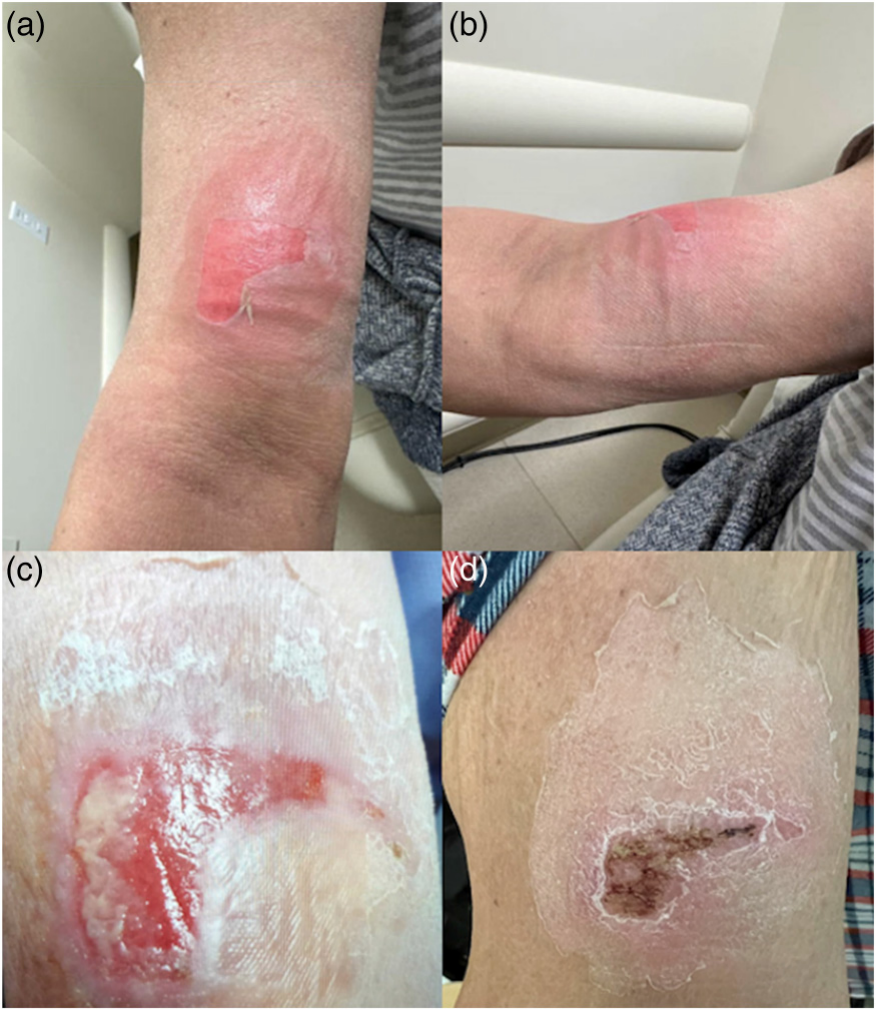
Case 1. Patient’s images of extravasation over time. 1a & 1b: 1 day after extravasation. Described as a smooth erythematous plaque with central areas of erosion and desquamation. 1c: 1 week after extravasation. Described as a scaly plaque with central erosion and fibrinous material. 1d: 2 weeks after extravasation. Described as a scaly patch with central hemorrhagic crusting in site of prior erosion.

On cycle 4, day 8 of EV therapy, the patient developed extravasation. EV infusion was given through a peripheral IV, which was placed in the left antecubital fossa. Pembrolizumab was not administered on that day. Nursing documentation did not note any symptoms of extravasation during or after the infusion. Upon returning home, the patient developed pain, erythema and a large blister at the infusion site, which intensified over the next several days and limited movement of her arm. After 3 days, she developed an erythematous plaque with drainage. She applied supportive measures including cold compresses, arm wrapping, and elevation as well as acetaminophen for pain relief. When seen for follow-up, physical examination was notable for a smooth erythematous plaque with central areas of erosion and desquamation (Figure 1(a) and (b)). In the next week, her swelling and erythema have progressively improved, but she continued to have drainage (Figure 1(c)). Given concern for superimposed cellulitis, she was prescribed clindamycin for 5 days. Her symptoms ultimately improved to grade 1 about 10 days after the initial extravasation (Figure 1(d)) and resolved during the following week. She then underwent chemotherapy port placement and was able to continue EV without further episodes of extravasation.

## Case # 2

A 63-year-old man underwent neoadjuvant chemotherapy with methotrexate, etoposide, Adriamycin, and cisplatin (MVAC) followed by radical cystectomy for muscle-invasive urothelial bladder cancer. He was found to have new retroperitoneal lymphadenopathy suspicious for cancer recurrence on surveillance imaging fourteen months post-surgery. He was initiated on atezolizumab 1200 mg every 3 weeks, but developed disease progression after 12 cycles of therapy. He was subsequently initiated on EV 1.25 mg/kg on days 1, 8, 15 out of a 28-day cycle.

**Figure 2.**
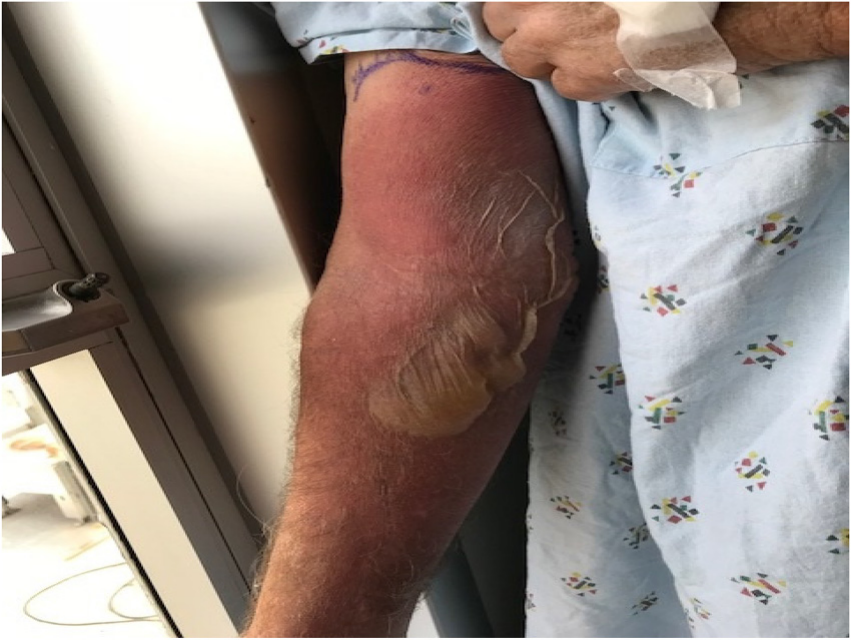
Case 2. Patient’s image of extravasation after 6 days.

On cycle 2, day 8 of EV therapy, the patient developed extravasation. EV infusion was given through a peripheral IV, which was placed in the right antecubital fossa. Intravenous access was initially obtained following five unsuccessful attempts. Nursing documentation did not note any symptoms of extravasation during or after the infusion. Three days later, the patient presented to the emergency room (ER) with right forearm erythema, edema, and severe pain that developed about 24 h following the infusion. Physical examination was notable a large flaccid blister on the right forearm (Figure 2) with exquisite tenderness to palpation. In the ER, he developed fever and altered mental status. He was initiated on vancomycin and ceftriaxone for cellulitis. After 3 days, he clinically improved, was transitioned to cefalexin, and discharged home. He returned to the ER 1 week after the extravasation due to continued arm pain. Physical exam was notable for a right erythematous bulla with central areas of erosion and desquamation. He was re-admitted for pain control and supportive care, but was discharged 4 days later. Three weeks after the extravasation, his symptoms improved to grade 1 and subsequently resolved. He subsequently underwent chemotherapy port placement and was able to continue EV without further episodes of extravasation.

## Discussion

Our patients both had a Naranjo Algorithm Assessment score of 7 notable for a “probable cause” of the adverse event.^3^ Drug extravasation can cause significant local tissue damage as well as superimposed infection and sepsis. Antineoplastic agents can be classified as neutrals, irritants, or vesicants. A neutral drug neither causes inflammation nor damage upon extravasation.^4^ An irritant is a drug that can cause inflammation, pain, or irritation at the extravasation site.^4^ In contrast, vesicants can result in tissue necrosis or formation of blisters.^4^ The mainstay of extravasation management consists of halting drug administration, attempting to aspirate the leaked volume of drug, photographing the site, elevating the limb, applying topical heat or cooling based on the antineoplastic agent that has extravasated, and thoroughly documenting the extravasation event.^5^ Extravasations of neutral or irritant agents are subsequently treated with additional supportive measures and close follow-up. In contrast, vesicants are more closely monitored as they have a potential for skin necrosis and compartment syndrome, with a low threshold for referral to plastic surgery.^5^ As new cancer therapies are being developed, it is imperative to classify them as irritants versus vesicants based on risk of tissue necrosis, as this will influence management of this adverse event.

EV is ADC that links a monoclonal antibody targeting Nectin-4 to monomethyl-auristatin E (MMAE), which is a microtubule inhibitor similar to taxane chemotherapies. EV is associated with a rare risk of extravasation, although the mechanism of associated tissue damage has not been well defined. Similar data with other ADCs are scarce and can be contradictory, as this is a fairly new class of antineoplastic agents. For instance, trastuzumab emtansine is an ADC targeting HER2 and is classified as an irritant, although some case reports have previously advocated for labeling it as a vesicant.^6,7^ Taxane chemotherapy agents including docetaxel and paclitaxel are considered vesicants, while albumin-bound nab-paclitaxel and cabazitaxel are considered indeterminant.^8^ Monoclonal antibodies are considered neutrals, but numerous case reports have described them as vesicants or irritants.^9,10^ Bispecific antibodies are a novel class of antineoplastic agents that simultaneously bind to two different targets and there is no data regarding their mechanism of tissue damage during extravasation. Based on the significant symptoms that occurred following EV extravasation in our patients, including large bullae and secondary cellulitis, we propose that EV acts as a vesicant upon extravasation.

Multiple guidelines addressing prevention of extravasation events are available, including from the European Oncology Nursing Society.^9^ Appropriate vascular access is imperative for infusions of antineoplastic agents. If peripheral access is used, larger caliber veins should be selected for IV placement since smaller veins may not withstand the flow and rate of an infusion.^11^ Choosing the best location for IV placement is also key. For instance, placement of IV access into the dorsum of the hand should be avoided as the veins are small, while antecubital fossa placement is also not ideal as extravasation events can go unnoticed. Additionally, it is important to continuously re-assess the administration site for any pain or swelling.^11^ In patients with borderline or poor IV access, particularly those who previously received IV antineoplastic therapies, central venous access should be obtained.

**Figure 3.**
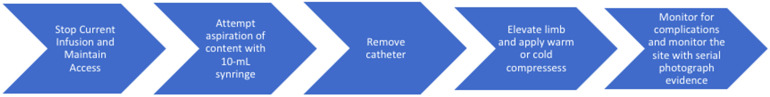
Timeline of management for extravasation events.

Understanding how to manage an extravasation event is crucial (Figure 3). If any pain or swelling is noted at the infusion site, the infusion should be immediately halted, while continuing to maintain IV access.^4,12^ There should be an attempt to aspirate the extravasated content with a 10 mL syringe and remove as much medication as possible.^9^ The catheter can then be removed and the site should be marked and photographed. Subsequent limb elevation can aid in reabsorption of the extravasated agent. Intermittent cold or warm compresses, depending on the type of agent responsible for extravasation, can be used to reduce pain and local inflammation.^9^ With taxane chemotherapy extravasation, dry warm compresses are recommended to be applied for 20 min four times daily for 1 to 2 days, as they can help with vasodilation and absorption of the agent.^9^ Further, cold compresses should be avoided following taxane extravasation since they can cause further tissue damage.^9^ Adequate follow-up to monitor patients for further sequela is necessary. If tissue necrosis or chronic ulceration occurs, it is imperative to promptly refer the patient to a plastic surgeon for potential surgical reconstruction and skin grafting.^13^ If superimposed infection develops, antibiotics should be initiated.

Proper documentation of an extravasation event is also essential, as extravasation events can result in serious injuries. Initial documentation can provide a baseline time and date of extravasation, the drug extravasated, initial signs and symptoms, photographic evidence, and management steps performed following extravasation. In both of our cases, that information would have been helpful in facilitating more prompt patient follow-up and possibly mitigating the subsequent sequela of the extravasations.

## Conclusions

Extravasation events can cause severe complications and lead to adverse outcomes. It is imperative to classify novel antineoplastic therapies as irritants or vesicants, which is frequently not done in the clinical trials that lead to approvals of these agents or in the preclinical setting. Based on the clinical picture of patients in this report, we propose that EV has properties of a vesicant. Providers must be familiar with techniques to prevent and manage extravasation events in order to minimize associated sequela for their patients.

## Supplemental Material

sj-png-1-opp-10.1177_10781552231185505 - Supplemental material for Drug extravasation with Enfortumab vedotin Click here for additional data file. Supplemental material, sj-png-1-opp-10.1177_10781552231185505 for Drug extravasation with Enfortumab vedotin by Christopher Ryan Grant, Dimitri de Kouchkovsky, Arash Rezazadeh Kalebasty and Nataliya Mar in Journal of Oncology Pharmacy Practice
