# Supplementary figures and images for: Drug extravasation with Enfortumab vedotin
Source: J Oncol Pharm Pract. 2023 Jul 4;29(7):1789–92. doi: 10.1177/10781552231185505 (PMC10612376; doi:10.1177/10781552231185505)

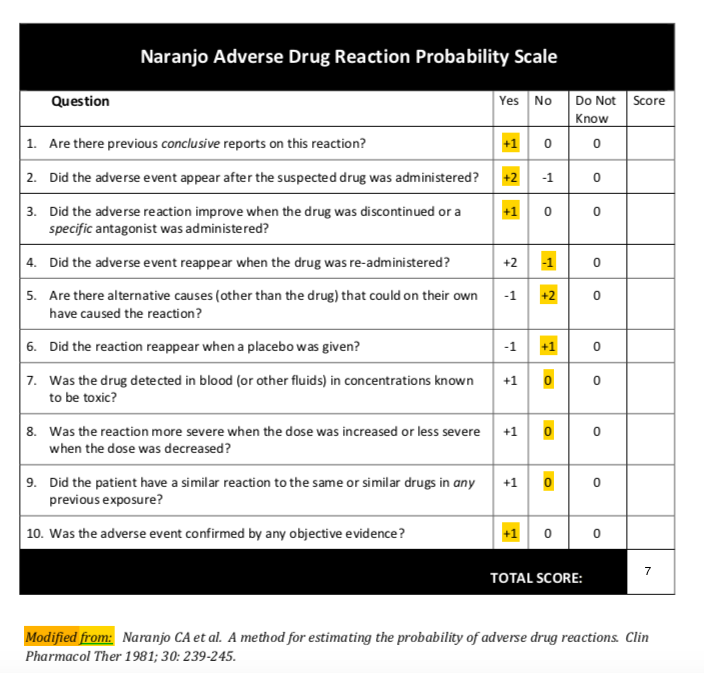

Supplement: sj-png-1-opp-10.1177_10781552231185505 - Supplemental material for Drug extravasation with Enfortumab vedotin [file sj-png-1-opp-10.1177_10781552231185505.png]
